# Impact of connecting tuberculosis directly observed therapy short-course with smoking cessation on health-related quality of life

**DOI:** 10.1186/1617-9625-10-2

**Published:** 2012-02-28

**Authors:** Ahmed Awaisu, Mohamad Haniki Nik Mohamed, Noorliza Mohamad Noordin, Abdul Razak Muttalif, Noorizan Abd Aziz, Syed Azhar Syed Sulaiman, Aziah Ahmad Mahayiddin

**Affiliations:** 1 College of Pharmacy, Qatar University, P.O. Box 2713, Doha, Qatar; 2 Faculty of Pharmacy, International Islamic University Malaysia, 25200 Kuantan, Malaysia; 3 Institute for Health Management, Ministry of Health, 59000 Kuala Lumpur, Malaysia; 4 Institute for Respiratory Medicine, 53000 Wilayah Persekutuan, Kuala Lumpur, Malaysia; 5 Faculty of Pharmacy, Universiti Technology Mara, 5600 Puncak Alam, Malaysia; 6 School of Pharmaceutical Sciences, Universiti Sains Malaysia, 11800 Penang, Malaysia

**Keywords:** DOTS, Outcomes, Quality of life, Smoking cessation intervention, Tuberculosis

## Abstract

**Background:**

With evolving evidence of association between tuberculosis (TB) and tobacco smoking, recommendations for the inclusion of tobacco cessation interventions in TB care are becoming increasingly important and more widely disseminated. Connecting TB and tobacco cessation interventions has been strongly advocated as this may yield better outcomes. However, no study has documented the impact of such connection on health-related quality of life (HRQoL). The objective of this study was to document the impact of an integrated TB directly observed therapy short-course (DOTS) plus smoking cessation intervention (SCI) on HRQoL.

**Methods:**

This was a multi-centered non-randomized controlled study involving 120 TB patients who were current smokers at the time of TB diagnosis in Malaysia. Patients were assigned to either of two groups: the usual TB-DOTS plus SCI (SCIDOTS group) or the usual TB-DOTS only (DOTS group). The effect of the novel strategy on HRQoL was measured using EQ-5D questionnaire. Two-way repeated measure ANOVA was used to examine the effects.

**Results:**

When compared, participants who received the integrated intervention had a better HRQoL than those who received the usual TB care. The SCIDOTS group had a significantly greater increase in EQ-5D utility score than the DOTS group during 6 months follow-up (mean ± SD = 0.98 ± 0.08 vs. 0.91 ± 0.14, *p *= 0.006). Similarly, the mean scores for EQ-VAS showed a consistently similar trend as the EQ-5D indices, with the scores increasing over the course of TB treatment. Furthermore, for the EQ-VAS, there were significant main effects for group [F (1, 84) = 4.91, *p *= 0.029, η^2 ^= 0.06], time [F (2, 168) = 139.50, *p *= < 0.001, η^2 ^= 0.62] and group x time interaction [F (2, 168) = 13.89, *p *= < 0.001, η^2 ^= 0.14].

**Conclusions:**

This study supports the evidence that an integrated TB-tobacco treatment strategy could potentially improve overall quality of life outcomes among TB patients who are smokers.

## Introduction

In recent years, there has been a global explosion of interest in the association between tuberculosis (TB) and tobacco smoking. Studies from different parts of the world have sufficiently documented consistent evidence that smoking substantially increases the risk of TB and death from it [1-4]. Tobacco smoking has in addition found to be significantly correlated with poor TB treatment outcomes and prognosis [5-8]. With such emerging evidence of association between the two diseases, recommendations for the inclusion of tobacco cessation interventions in TB care are becoming increasingly important and more widely disseminated [9-13]. Therefore, the integration of tobacco control with TB control programs is an important global agenda of public health significance. Patients undergoing TB treatment under the directly observed therapy, short-course (DOTS) are typically in regular contact with the healthcare provider for a minimum duration of six months. At every encounter with their clients or patients, healthcare providers in TB care settings have a unique opportunity to deliver smoking cessation intervention (SCI). It is expected that patients may be more amenable to health education messages when they are ill and such periods may be teachable moments for behavioral changes [11,14,15]. Therefore, there are opportunities for tobacco cessation interventions among TB patients that are often grossly underutilized.

El Sony and colleagues have examined the feasibility of introducing a brief tobacco cessation intervention for new cases of pulmonary TB in Sudan [16]. The study reported that healthcare providers had the willingness to provide tobacco cessation counseling to TB patients and that the intervention was feasible and effective for those enrolled within routine TB service [16]. Following this pioneering and landmark study by El Sony and associates, the International Union Against Tuberculosis and Lung Disease (IUATLD) designed an educational series on the provision of tobacco cessation interventions for TB patients [14,15,17-21]. In this series, the authors strongly advocated for the inclusion of brief smoking cessation advice in standard TB case management [14,15].

Other additional giant efforts along this direction have been made by the IUATLD and the World Health Organization (WHO), leading to the development of two comprehensive practice and policy guidelines on tobacco cessation interventions in TB programs [22,23]. Currently, pilot projects are ongoing in a number of countries around the world. For instance, smoking cessation service has been introduced in TB care management in Rio de Janeiro, Brazil [24]. Similar services have also been introduced through the Practical Approach to Lung Health (PAL) at pilot sites in Indonesia, Nepal, Kyrgyzstan, and Egypt [24]. What remains to be demonstrated, however, are the impacts of such services on treatment outcomes and health-related quality of life (HRQoL).

HRQoL is increasingly been used as an outcome measure for assessing disease treatment and it is essential in evaluation of new interventions or therapies [25]. In the present study, we utilized the EuroQoL five-dimension questionnaire (EQ-5D) to assess TB patients' self-evaluations of the impact of the disease and the associated treatments on their physical, mental, and social well-being and functioning. EQ-5D was selected because of its track record of excellent psychometric properties and it appeared to be a valid and reliable tool in TB setting. Dion et al. have evaluated the feasibility and reliability of EQ-5D among TB patients [26]. The instrument also has the advantage of brevity and been linguistically validated in all the commonly spoken languages of the country of study (Malay, Chinese, and Malaysian English). The primary aim of the current research was to evaluate the impact of adding tobacco cessation intervention to conventional DOTS for TB on clinical and quality of life outcomes of patients undergoing treatment for TB. Here, we report the potential impact of the value added TB-tobacco treatment strategy on HRQoL.

## Materials and methods

### Study design

A multi-centered prospective non-randomized controlled trial using quasi-experimental design was conducted in Malaysia. Using Transtheoretical Model approach [27], 120 eligible participants who were current smokers at the time of TB diagnosis were assigned to either of two treatment groups: the usual TB-DOTS plus SCI (SCIDOTS group) or the usual TB-DOTS alone (DOTS group).

**Figure 1:**
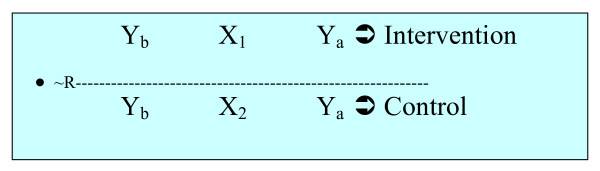
**Study design for the integrated TB-tobacco intervention study**.

The study design can be schematically represented as below:

In Figure 1 above, ~R signifies that there was no randomization of subjects into the treatment groups. Y_b _represents the baseline HRQoL outcome measures (before the interventions were initiated), whereas Y_a _implies the HRQoL outcomes at different periods during the interventions. X_1 _and X_2 _represent the integrated intervention and the usual TB care, respectively.

### Study area and setting

This study was conducted at five chest clinics in Malaysia. The selected clinics were considered as TB referral centers from which all diagnosis or confirmation of TB cases must come from among the population they serve. The research was reviewed and approved by the Medical Research Ethics Committee of the National Institutes of Health, Malaysia in April 2008.

### Study sample and sample size determination

The sample for the present study included all current cigarette smokers newly diagnosed with TB between June 2008 and April 2009. Patients were referred at the point of initiating DOTS regimen by attending physicians to TB DOTS providers who were purposely trained to provide tobacco cessation intervention in TB care settings [13]. Patients were recruited into the study upon fulfillment of eligibility criteria and written informed consent. Power analysis and sample size requirements in this study were predicated on the primary outcome measure, that is the overall TB treatment outcome based on comparison of TB cure rates between the two treatment groups. A statistical power of 80% to detect a moderate effect size (20% difference in cure rates) at a 95% confidence level were applied in the sample size determination. By WHO standard of care, DOTS has a target to achieve a 95% cure rate in previous reports; therefore, we assumed a cure rate of 95% and 75% in the intervention and the control groups, respectively. The minimum sample size was estimated as 48 per treatment arm with a 2-sided alpha level of 0.05. We assumed a dropout rate of 25%, giving rise to 60 participants to be recruited per each treatment arm.

### Intervention

The participants in both the SCIDOTS and the DOTS groups went to the clinics on daily-basis to receive TB DOTS for at least 6 months. Eleven sessions of individualized cognitive behavioral therapy (CBT) with or without nicotine replacement therapy (NRT) were offered to each participant in the SCIDOTS group, while the DOTS group received the same number of sessions of usual counseling for TB. The follow-up clinic appointments for SCI (beginning on the quit date) were as follows: weekly for the first month, fortnightly for the second and third month, and monthly from the fourth to the sixth month. HRQoL outcomes were measured by using EQ-5D and the effects of the novel strategy on these were determined by comparing the two groups.

**Figure 2:**
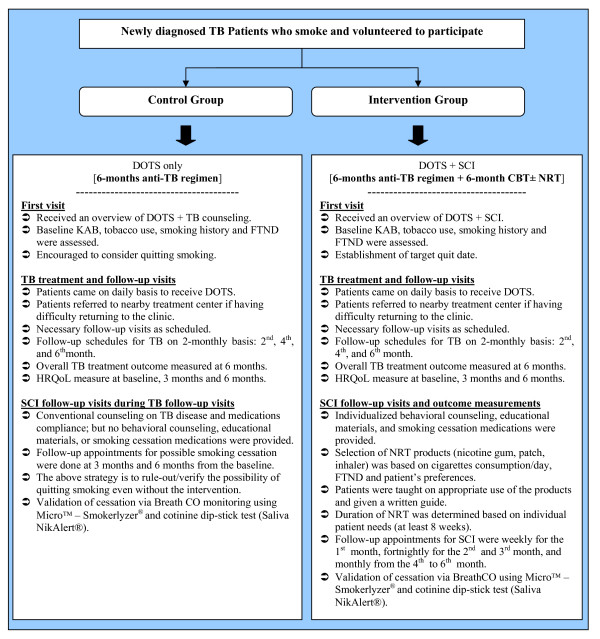
**Flowchart of the interventions used in the study (usual care vs. integrated intervention groups)**.

A summary of the interventions provided and outcomes measurements for each of the two treatment groups is provided in Figure 2.

### Study instrument and HRQoL outcome measures

The EQ-5D questionnaire was used for measuring the quality of life of the study participants. The instrument was subjected to standardized linguistic validation processes for use in Malaysian population by EuroQoL Group, UK. Permission was obtained prior to use in this study. HRQoL outcomes were measured periodically (at baseline, at 3 months, and at 6 months of TB treatment) for all participants using the EQ-5D questionnaire, which consists of two sections: the EQ-5D self-descriptive assessment and the visual analogue scale (EQ-VAS) [28-30]. The EQ-5D descriptive system comprises of five domains (mobility, self-care, usual activities, pain/discomfort, and anxiety/depression), each having three response choices: no problems, moderate problems, and severe problems. Participants were asked to indicate their own health state by ticking in the box against the most appropriate statement. Participants' health state classifications in the descriptive system were converted into composite utility scores using the UK social tariff [31,32]. In addition, patients also self-rated their health by drawing a line to the EQ-VAS to represent their current health status. EQ-VAS rates the current health status from 0 to 100, where the endpoints are labeled as best imaginable health state (100) and worst imaginable health state (zero).

### Statistical analyses

The data were analyzed by using SPSS version 16.0 (SPSS Inc., Chicago, IL) and the level of statistical significance was set *a priori *at *p *< 0.05. Homogeneity of baseline data was determined by using two-sample tests including Student's *t*-test for normally distributed continuous variables, and Pearson's chi-square (χ^2^) or Fisher's exact test for categorical variables. In the analysis of HRQoL, each domain of the EQ-5D self-descriptive assessment was categorized into "no problem", "moderate problem", and "severe problem" and the data were presented as frequencies and percentages. χ^2 ^or Fisher's exact tests were used to compare between the SCIDOTS and the DOTS groups for each specific domain. The EQ-5D health state classification for each subject was converted into an overall utility score [31,32]. Means and standard deviations for EQ-5D utility indices and EQ-VAS scores were calculated and tabulated. Independent *t*-test was employed to examine the effects of group on HRQoL (measured using the EQ-5D utility and EQ-VAS scores) from baseline to the end of the 6-month follow-up. Furthermore, two-way repeated measure ANOVA was used to examine the main effect of group (intervention and control), time (baseline, 3 months, and 6 months), and group x time interaction on EQ-5D utility scores and EQ-VAS. Sphericity-assumed estimates were applied if Mauchly's test indicated no violation of assumptions; otherwise, Greenhouse-Geisser estimates were used.

## Results

The similarity of the study groups with respect to socio-demographic, disease- and smoking-related characteristics was compared. This was done in order to ascertain whether the groups were similar in related characteristics, despite absence of randomization in the design of the study. From the results obtained, subjects in the two groups were homogenous with respect to most of the characteristics [33]. Although participants in the control group were older than those in the intervention group (45.8 ± 14.4 years vs. 41.6 ± 14.4 years), this difference was not statistically significant (t = -1.334, *p *= 0.186). The groups did not differ significantly in relation to gender (Fisher's Exact *p *= 1.000), ethnicity (Fisher's Exact *p *= 0.431), and marital status (Fisher's Exact *p *= 0.961). In both groups, TB patients smoked an average of 16 cigarettes per day. Similarly, during diagnosis, the clinical presentations of TB disease were similar between the treatment groups. Furthermore, the distribution of other baseline data was not different between the intervention and the control groups. Hence, the effect of the integrated TB-tobacco intervention on HRQoL and other outcomes was determined.

### Tobacco cessation and TB treatment outcomes

Results related to tobacco cessation outcomes (i.e. quitting smoking) were previously published elsewhere [33]. Abstinence was biochemically validated using exhaled CO and saliva cotinine testing. The study recorded a linear trend over time on 7-day point prevalence abstinence (PPA) and continuous abstinence (CA) in the SCIDOTS group, whereas abstinence rates remained fairly constant in the usual care (DOTS) group. At the sixth month of follow-up, patients who received the integrated intervention had a significantly higher rate of success in quitting smoking when compared with those who received the usual TB care alone (78% vs. 9%, respectively; *p *< 0.001). Further details on smoking cessation and TB treatment outcomes could be found in the study published by Awaisu et al. (2011).

The key findings in relation to HRQoL are broadly classified under the two subheadings below.

### EQ-5D self-descriptive assessment by dimension

Table 1 presents a comparison of self-description assessment between the intervention and the control groups in five domains. The Majority of the participants in both groups reported no problems with mobility and self-care for all time periods. The trends in changes observed over time in these two dimensions were also similar between the two treatment groups. Moreover, there were no significant differences between participants in the SCIDOTS and the DOTS groups in their self-rating of mobility and self-care at baseline, 3 months and 6 months (Table 1).

**Table 1:** Comparison of EQ-5D self-description assessment between control (DOTS) and intervention (SCIDOTS) groups^a^

Dimension	Assessment	Baseline			End of 3 months			End of 6 months		
		**DOTS (n = 46)**	**SCIDOTS (n = 40)**	***p*-value**	**DOTS (n = 46)**	**SCIDOTS (n = 40)**	***p*-value**	**DOTS (n = 46)**	**SCIDOTS (n = 40)**	***p*-value**

	No problem	32 (69.6%)	27 (67.5%)		43 (93.5%)	37 (92.5%)		43 (93.5%)	39 (97.5%)	
MOBILITY	Moderate problem	13 (28.3%)	13 (32.5%)	0.603	3 (6.5%)	3 (7.5%)	1.000	3 (6.5%)	1 (2.5%)	0.620
	Severe problem	1 (2.2%)	0 (0)		0 (0)	0 (0)		0 (0)	0 (0)	

	No problem	34 (73.9%)	29 (72.5%)		43 (93.5%)	36 (90.0%)		44 (95.7%)	39 (97.5%)	
SELF-CARE	Moderate problem	11 (23.9%)	11 (27.5%)	0.612	3 (6.5%)	4 (10.0%)	0.700	2 (4.3%)	1 (2.5%)	1.000
	Severe problem	1 (2.2%)	0 (0)		0 (0)	0 (0)		0 (0)	0 (0)	

	No problem	31 (67.4%)	18 (45.0%)		37 (80.4%)	34 (85.0%)		35 (76.1%)	38 (95.0%)	
USUAL ACTIVITIES	Moderate problem	12 (26.1%)	22 (55.0%)	0.011	9 (19.6%)	5 (12.5%	0.395	11 (23.9%)	2 (5.0%)	0.015^b^
	Severe problem	3 (6.5%)	0 (0)		0 (0)	1 (2.5%)		0 (0)	0 (0)	

	No problem	16 (34.8%)	13 (32.5%)		34 (73.9%)	33 (82.5%)		40 (87.0%)	40 (100.0%)	
PAIN	Moderate problem	27 (58.7%)	27 (67.5%)	0.234	11 (23.9%)	7 (17.5%)	0.474	6 (13.0%)	0 (0)	0.028
	Severe problem	3 (6.5%)	0 (0)		1 (2.2%)	0 (0)		0 (0)	0 (0)	

	No problem	20 (43.5%)	17 (42.5%)		36 (78.3%)	35 (87.5%)		41 (89.1%)	38 (95.0%)	
ANXIETY	Moderate problem	24 (52.2%)	21 (52.5%)	0.988	9 (19.6%)	4 (10.0%)	0.466	5 (10.9%)	2 (5.0%)	0.442
	Severe problem	2 (4.3%)	2 (5.0%)		1 (2.2%)	1 (2.5%)		0 (0)	0 (0)	

^a^Fisher's Exact and ^b^Chi-square (χ^2^) tests were applied in calculating *p*-values

Conversely, substantial proportions of the TB patients believed that they had moderate to severe problems in relation to usual activities, pain/discomfort, and anxiety/depression at diagnosis. At baseline, 12 patients (26.1%) in the usual care group rated themselves as having moderate problems with usual activities compared with 22 patients (55.0%) in the integrated intervention group (*p *= 0.011). Interestingly, at six months, the proportion of patients in the SCIDOTS group who had no problems with this dimension was significantly higher than that of patients in the DOTS group (95.0% vs. 76.1%; *p *= 0.015).

Furthermore, about two-thirds of the study participants in both groups assessed themselves as having moderate to severe pain or discomfort during TB diagnosis (*p *= 0.234). This problem waned over time and at the end of 6 months of treatment, all patients (100%) in the integrated intervention group versus 87% in the usual care group admitted that they experienced no pain or discomfort (*p *= 0.028). A summary of these findings is presented in Table 1. Similarly, during diagnosis, about 52% of the TB patients in both arms considered themselves as moderately anxious or depressed. Having problems in this dimension progressively decreased by about two to four folds during each visit.

### EQ-5D utility and EQ-VAS scores

The means and standard deviations for EQ-5D utility indices and EQ-VAS scores were computed and summarized in Table 2. There was no significant difference at baseline between the SCIDOTS and the DOTS groups for mean EQ-5D utility score (0.72 ± 0.20 vs. 0.70 ± 0.31, t = 0.37, *p *= 0.715). The integrated intervention group had a greater, but statistically non-significant increase in EQ-5D utility index when compared with the usual care group at 3 months follow-up (mean = 0.91 ± 0.16 vs. 0.87 ± 0.22, t = 0.98, *p *= 0.333). However, at 6 months follow-up, the former had significantly greater increase in EQ-5D utility score than the latter (mean = 0.98 ± 0.08 vs. 0.91 ± 0.14, t = 2.85, *p *= 0.006). Similarly, the mean scores for EQ-VAS showed a closely similar trend as the EQ-5D utility indices, with the scores increasing over the course of TB treatment (Table 2).

**Table 2:** Health-related quality of life outcomes according to group assignment^a^

HRQoL outcome measure	Control Group (DOTS)n = 46	Intervention Group(SCIDOTS)n = 40	t (df = 84)	p-value^b^
**EQ-5D utility index**				
Baseline	0.70 ± 0.31	0.72 ± 0.20	0.37	0.715
End of 3-months follow up	0.87 ± 0.22	0.91 ± 0.16	0.98	0.333
End of 6-months follow up	0.91 ± 0.14	0.98 ± 0.08	2.85	0.006
**EQ-VAS**				
Baseline	57.04 ± 12.74	55.00 ± 13.83	-0.71	0.478
End of 3-months follow up	70.50 ± 13.62	73.60 ± 13.15	1.07	0.288
End of 6-months follow up	73.87 ± 12.01	87.28 ± 11.44	5.26	< 0.001

**^a^**Plus-minus values are means ± SD; **^b^***p*-values were calculated with the use of independent t-test for all continuous data reporting mean values

*EQ-5D *EuroQoL 5 dimensions; *VAS *visual analogue scale

Two-way repeated measure ANOVA was used to examine the main effect of group, time, and group x time interaction on EQ-5D utility score and on EQ-VAS score as shown in Table 3. Covariates were not used in the analyses despite significant baseline differences between the SCIDOTS and the DOTS groups in nicotine dependence [Fagerström test for nicotine dependence (FTND) score] and in expired CO level, as there was no significant correlation between the baseline FTND score and the baseline EQ-5D utility index (r = -0.11, *p *= 0.3); and between the baseline CO level and baseline EQ-5D utility index (r = -0.21, *p *= 0.06). Mauchly's test of sphericity showed that the assumption of sphericity was violated (χ^2 ^= 20.43, *p *< 0.001); thus, Greenhouse-Geisser estimates were applied. There were no significant main effects for group [F (1, 84) = 1.86, *p *= 0.176, η^2 ^= 0.02] and group x time interaction [F (2, 138) = 0.548, *p *= 0.545, η^2 ^= 0.006], but there was significant main effect for time [F (2, 138) = 49.42, *p *< 0.001, η^2 ^= 0.37].

**Table 3:** Two-way repeated measure ANOVA summary for EQ-5D utility index and EQ-VAS

EQ-5D utility index						
**Source**	**Type III sum of squares**	**Df**	**Mean square**	**F**	*p*-value*	**Partial Eta squared**

**Group**	.128	1	.128	1.864	.176	.022
**Error **(between subjects)	5.789	84	.069			
**Time (trial) for EQ-5D utility score**	2.592	1.642	1.579	49.421	.000	.370
Group*Time	.029	1.642	.018	.548	.545	.006
**Error **(within subjects)	4.406	138	.026			

**EQ-VAS**						

**Source**	**Type III sum of squares**	**Df**	**Mean square**	**F**	*p*-value*	**Partial Eta squared**

**Group**	1491.599	1	1491.599	4.906	.029	.055
**Error **(between subjects)	25538.509	84	304.030			
**Time (trial) for EQ-VAS**	26594.807	2	13297.404	139.50	.000	.624
Group*Time	2648.218	2	1324.109	13.891	.000	.142
**Error **(within subjects)	16013.696	168	95.320			

Similarly, the main effects of group, time, and group x time interaction on EQ-VAS as shown in Table 3 were assessed. Assumption of sphericity was met as indicated by Mauchly's test of sphericity (χ^2 ^= 2.5, *p *= 0.287); thus, sphericity-assumed estimates were applied. There were significant main effects for group [F (1, 84) = 4.91, *p *= 0.029, η^2 ^= 0.06], time [F (2, 168) = 139.50, *p *< 0.001, η^2 ^= 0.62] and group x time interaction [F (2, 168) = 13.89, *p *< 0.001, η^2 ^= 0.14]. In addition, more in-depth analyses using pairwise comparison showed significant mean differences across all levels: level 1 vs. level 2 (∆mean = 16.03, *p *< 0.001); level 1 vs. level 3 (∆mean = 24.55, *p *< 0.001) and level 2 vs. level 3 (∆mean = 8.52, *p *< 0.001).

## Discussion

This pilot project has demonstrated the potential advantages of connecting tobacco dependence treatment with TB treatment in improving quality of life outcomes. Our findings suggest that the combination of DOTS for TB and SCI via CBT and pharmacotherapy has a great potential to yield better treatment outcomes among TB patients who smoke [4,10,11,34]. The EQ-5D classifier indicated that the usual activities, pain/discomfort, and anxiety/depression domains seemed to be more affected during diagnosis than other domains. Improvements in all of the five dimensions of EQ-5D questionnaire were however observed over time in both the integrated intervention and the usual care groups. The participants had shown improvements in both physical and mental functioning. Moreover, no differences were observed between the two groups in terms of mobility, self-care, and anxiety/depression throughout the follow-up periods.

During the last follow-up visit, however, significantly greater proportions of participants enrolled in the SCIDOTS group rated themselves better from the perspective of usual activities and pain/discomfort when compared with the DOTS group. In our opinion, pain in the context of this study may be rather subjective than a real physical or bodily pain, as TB is not usually associated with any physical injury or disability and the majority of the participants did not have such abnormalities. The improvements observed in pain/discomfort domain could possibly be contributed by the corresponding improvements in anxiety/depression.

In summary, the findings of the current study have demonstrated the positive impacts of TB treatment, especially when combined with SCI on self-descriptive assessment of current health status. Previous studies have documented that many aspects of TB along with its treatment could potentially compromise patients' HRQoL [35-37]. Clinical factors were observed to correlate with poorer HRQoL among TB patients. Some of the factors reported to influence quality of life among TB patients include disease duration, number of symptoms before treatment, reactivation of previous TB infection, hemoptysis, hospitalization, underlying chronic conditions, anemia, and baseline white blood cells counts [38,39]. In the present study, most of the socio-demographic and clinical characteristics were similar between the two treatment groups at baseline; therefore, it is unlikely that the differences observed in HRQoL were due to variations in these variables.

Likewise, the HRQoL of TB smokers measured using both EQ-5D utility scores and EQ-VAS was relatively low at baseline (before initiation of treatment), signifying a decline in HRQoL [36,37,39]. We believe that the decline seen in the quality of life of TB smokers may probably be multidimensional and contributed by other factors as discussed previously. Consistent with the 5-item descriptive system, the EQ-VAS score and the EQ-5D utility index both increased consistently over time, suggesting that patients generally continued to improve over time during TB treatment. These observations strongly support previous studies of increased quality of life for patients receiving TB treatment [37,38,40].

The *a priori *assumption that participants in the integrated intervention arm would have greater increases in HRQoL during 3 months and 6 months follow-up assessments was tested using two-way repeated measure ANOVA. Over the TB treatment period, higher EQ-5D utility score gains and EQ-VAS score gains were observed among patients who received the SCIDOTS intervention compared to those who received DOTS only, especially at 6 months. Overall, the anti-TB treatment, especially when combined with tobacco cessation appeared to have a positive and greater impact on improving patients' HRQoL. Moreover, the physical health of the participants tended to recover more quickly than their mental well-being. Despite heterogeneity in measuring instruments, these findings are consistent with other quality of life studies in TB setting [38,40]. However, the findings should be interpreted with the notion that ceiling and floor effects are a common problem for the application of health utility instruments in TB [35].

It is also worthwhile to highlight nicotine withdrawal syndrome as an important confounder in the anxiety/depression domain of the EQ-5D. Participants who received the SCI tended to present with nicotine withdrawal symptoms including anxiety and depression [41,42]. This may invariably have an overall net effect on the quality of life. This implies that the quality of life of the intervention group could have even been better than it were.

The major strength of the current study is the application of an intensive tobacco cessation intervention; using a combination of pharmacotherapy and CBT rather than brief intervention in the evaluation of the impact of an integrated tobacco cessation intervention in TB care setting on treatment outcomes and quality of life. The study has also utilized biochemical measures rather than self-reporting alone in the validation of smoking abstinence. Moreover, it has expanded its scope to investigate humanistic outcomes, particularly HRQoL.

Nevertheless, there were some limitations to the study. Most notably, the study used a non-randomized controlled design. The non-randomized approach to group assignment was in the light of the nature of nicotine addiction, which necessitated us to adopt the Transtheoretical Model of Stages of Change during the assignment of subjects into the treatment groups. Consequently, there were inherent potential threats to internal validity such as selection bias, because assignment to groups was based on individual participant's decision.

## Conclusion

The present study has demonstrated a potential positive impact of connecting smoking cessation to DOTS, with documented effects on overall HRQoL of TB patients who smoke. The findings suggest that the integrated approach may confer advantages on HRQoL on short- and long-term. The lessons learnt from this pilot study can be applied to expansion and scale-up projects in the future. Future studies should evaluate the long-term benefits of the integrated TB-tobacco intervention approach on several outcomes of interest.

## Competing interests

The authors declare that they have no competing interests.

## Authors' contributions

AA and MHNM conceived the original research idea, designed the study and wrote its protocols. AA, MHNM, NMN, ARM, NAA, SASS, and AAM, all contributed in the proposal writing. AA, MHNM, NMN, NAA, SASS, ARM and AAM all participated in the collection, analysis and interpretation of the data. AA wrote the initial and final drafts of the manuscript. MHNM, NMN, NAA, SASS, ARM and AAM substantially helped in improving the intellectual contents and scientific merit of the entire manuscript. All authors read and approved the final manuscript.

## Funding

This project was largely funded by a research grant provided by the Institute for Health Management, National Institutes of Health, Ministry of Health, Malaysia.
